# Comments on “High Altitude Pulmonary Edema in an Experienced Mountaineer. Possible Genetic Predisposition”

**DOI:** 10.5811/westjem.2015.6.27505

**Published:** 2015-10-20

**Authors:** Gaurav Sikri

**Affiliations:** Armed Forces Medical College, Department of Physiology, Pune, India

To the Editor:

I read with interest the case report by Whitlow and Davis in the November 2014 issue of the Western Journal of Emergency Medicine regarding management of high altitude pulmonary edema (HAPE) in an experienced mountaineer.^1^ The authors have appropriately highlighted the need of descent and supplemental oxygen for treating HAPE, a potentially fatal disease if left untreated. The patient in this study, a 25-year-old sea-level resident, was diagnosed as a case of HAPE on the basis of history of acute ascent to 3200m and onset of symptoms and signs suggestive of HAPE within 72 hours of high altitude exposure. He was treated with 100% oxygen, albuterol and ipratropium nebulizers, inhaled and intravenous dexamethasone, intravenous hydralazine, and intravenous furosemide. Subsequently, with improvement of symptoms, he was continued on intravenous dexamethasone. However, as per the Wilderness Medical Society (WMS) evidence-based guidelines of 2009 for clinicians for prevention and treatment of acute altitude illness, diuretics have no role to play in the treatment of HAPE, as these patients are likely to have co-existing intra-vascular volume depletion.^2^ Moreover, dexamethasone is not recommended for treatment of all cases of HAPE and it has only a preventive role in HAPE-susceptible individuals.^2^ Analysis of the history and examination of this patient reveals that a differential diagnosis of asthma was considered along with HAPE. As a reader, I was inquisitive to know if an X-ray facility was available at the community academic emergency department, and if so, an X-ray chest of the patient on arrival would have helped in confirming the diagnosis of HAPE.
